# Recommendations for assessing motor performance in individuals with dementia: suggestions of an expert panel – a qualitative approach

**DOI:** 10.1186/s11556-019-0212-7

**Published:** 2019-04-13

**Authors:** Sandra Trautwein, Bettina Barisch-Fritz, Andrea Scharpf, Willem Bossers, Marcus Meinzer, Simon Steib, Thorsten Stein, Klaus Bös, Alexander Stahn, Claudia Niessner, Stefan Altmann, Rita Wittelsberger, Alexander Woll

**Affiliations:** 1 Institute of Sports and Sports Science, Karlsruhe Institute of Technology, Engler-Bunte-Ring 15, 76131 Karlsruhe, Germany; 2 Center for Human Movement Sciences, University Medical Center Groningen, University of Groningen, Groningen, The Netherlands; 3 Centre for Clinical Research, University of Queensland, Brisbane, Australia; 4 Department of Neurology, Greifswald University Medicine, Greifswald, Germany; 5 Department of Sport Science and Sport, Friedrich-Alexander University Erlangen-Nürnberg, Erlangen, Germany; 6 Unit of Experimental Psychiatry, Department of Psychiatry, Perelman School of Medicine at the University of Pennsylvania, Philadelphia, USA; 7 Institute of Physiology, Charité – Universitätsmedizin Berlin, a corporate member of Freie Universität Berlin, Humboldt-Universität zu Berlin, and Berlin Institute of Health, Berlin, Germany

**Keywords:** Dementia, Physical activity, Exercise, Motor performance, Motor assessments

## Abstract

**Background:**

Recommendations for assessing motor performance in individuals with dementia (IWD) are rare, and most existing assessment tools previously applied in IWD were initially developed for healthy older adults. However, IWD and their healthy counterparts differ in motor and cognitive capabilities, which needs to be considered when designing studies for this population. This article aims to give recommendations for motor assessments for IWD and to promote standardisation based on a structured discussion of identified assessment tools used in previous trials.

**Methods:**

Appropriateness and standardisation of previously applied motor assessments for IWD were intensively discussed using a qualitative approach during an expert panel. Furthermore, the use of external cues and walking aids, as well as psychometric properties were considered. Starting with a comprehensive overview of current research practice, the discussion was gradually specified and resulted in the elaboration of specific recommendations.

**Results:**

The superior discussion emphasised the need for tailoring motor assessments to specific characteristics of IWD and attaching importance to standardised assessment procedures. Specific recommendations include the use of sequential approaches, which incorporate a gradual increase of complexity from simple to more difficult tasks, a selection of motor assessments showing sufficient relative reliability and appropriateness for IWD, as well as allowing external cues and walking aids when restricted to repeated instructions and commonly used devices, respectively.

**Conclusions:**

These are the first recommendations for assessing motor performance in IWD based on a comprehensive qualitative approach. Due to limited evidence, it was not possible to address all existing questions. It is therefore important to evaluate these recommendations in studies with IWD. Besides tailoring and evaluating available assessments, future research should focus on developing specific tools for IWD. Moreover, further progress in standardisation is necessary to enhance comparability between different trials. This article provides initial approaches for overcoming existing limitations in trials with IWD by giving recommendations and identifying future research questions, and therefore contributes to enhancing evidence regarding efficacy and effectiveness of physical activity interventions.

## Background

Designing studies to investigate the efficacy and effectiveness of physical activity on motor and cognitive performance in individuals with dementia (IWD) is challenging. Despite increasing research in this area [1], there still is insufficient evidence, which can be explained by methodological limitations, unspecific interventions, or inappropriate assessments [2–4]. This emphasises the need for further high quality studies guided by suggestions for optimised interventions and sensitive assessment tools.

Previous trials which aimed at improving motor performance in IWD frequently applied interventions and assessment tools not adapted to the target population [2, 5]. Considering significant differences in cognitive and motor performance [6–10], however, it is not possible to directly translate study designs developed for healthy older adults to IWD. Especially interventions and assessments need to be tailored to the specific characteristics of IWD, such as decreased executive functioning, reduced attentional and memory capacities [6, 7], diminished ability to develop and perform new complex motor sequences [11], and impaired gait and balance performance [8, 10]. For motor assessments, it is important to consider these cognitive and motor impairments, as both aspects hamper a successful participation. Besides research, this is also important for clinical practice considering prognostic, diagnostic, and therapeutic reasons [12, 13].

Due to cognitive impairment, IWD are often not able to concentrate on comprehensive or complex, unknown tasks, and frequently show difficulties in comprehending instructions, developing appropriate motor actions, and remembering these during execution [11]. Considering this, there are indications that cognitive impairment may influence the outcome of motor assessments and compromise their feasibility [14]. Appropriate assessments therefore need to be tailored to cognitive impairments. It has been suggested that assessments should be of short duration, use appropriate instructions (e.g. no verbal focus, demonstration of the task, clear, short, and repeated instructions [11]), and include simple motor tasks. Moreover, previous studies discuss the use of external cues thereby considering decreased cognitive abilities. For example, van Iersel et al. [15] emphasise the need for external cues, which are required to ensure feasibility and to achieve high relative reliability. In contrast, Hauer et al. [11] argue that external cues could influence the results and possibly reflect speed, reliability, and quality of external cues rather than actual performance of IWD.

Considering motor influences, disease-specific gait and balance impairments are often accompanied by age-related degeneration, such as frailty [16]. Together, they frequently compromise the ability of IWD to walk independently, to complete more complex balance tasks, and to cover longer distances [10, 17], which are necessary to participate in many assessments. Thus, appropriate assessments also need to be tailed to motor impairments. In this context, it is important to discuss the use of walking aids during assessments. IWD are frequently dependent on walking aids [15], which ensure participants’ safety. However, an investigation in geriatric patients without or with mild to moderate cognitive impairment found that walking aids impede the detection of gait or mobility deficits and thus adversely affect identifying changes over time [18].

Furthermore, the significance of assessments depends on sound psychometric properties, which are necessary to draw meaningful conclusions [5]. Investigations determining psychometric properties of motor assessment tools in IWD are rare, especially concerning validity [19]. Considering test-retest reliability, intraclass correlation coefficients ranging between .42 and .99 and high intra-individual variability were found for most previously applied assessment tools, indicating fair to excellent relative reliability but insufficient absolute reliability [15, 20–30]. Summarising these results, it has been concluded that the considered motor assessments are appropriate for detecting inter-individual differences in cross sectional or controlled intervention studies, whereas the intra-individual variability was too large to be suitable for investigating intra-individual performance changes [20, 21].

Considering all above-mentioned aspects leads us to the question which motor assessments are actually appropriate for IWD. Unfortunately, there is currently a lack of recommendations or guidelines on how to assess motor performance in IWD. In two of the few studies addressing this issue, Bossers et al. [19] and McGough et al. [31] performed a systematic and scoping review, respectively, and recommended using the Berg Balance Scale, the Performance Oriented Mobility Assessment, the Timed Up & Go Test (TUG), short distance walk tests (WT), sit-to-stand (STS) tests, isometric strength assessments, and the 6-min WT for IWD in mild to moderate stages of the disease. Both research groups derived their recommendations based on frequency of use and observed outcome effect sizes in previous trials. They also considered investigations of psychometric properties. However, the authors noted large heterogeneity in the assessment tools used across trials and indicated that insufficient information about feasibility, sensitivity to change, and psychometric properties was frequently provided.

These systematic reviews provide first indications for appropriate motor assessment tools for IWD. However, these recommendations predominantly concern quantitative aspects, and subsequently do not sufficiently consider specific characteristics. There are no comprehensive qualitative approaches focusing on tailoring motor assessments to IWD.

Besides tailoring motor assessments to the specific characteristics of IWD, their standardisation is also important. Gonçalves et al. [4] note that large heterogeneity in outcomes and assessment tools limits evidence of the efficacy and effectiveness of physical activity in IWD. A detailed review of previous trials also shows that different variations of motor assessment tools were used, which hampers comparability. This emphasises the urgent need to standardise existing motor assessments used in IWD.

This article describes the outcome of a consensus meeting of an international expert panel that aimed to derive recommendations for assessing motor performance in IWD. Due to limited information available for appropriate assessments and their standardisation, the specific goals of the panel were:

1. To discuss the selection of appropriate existing motor assessments, and the standardisation of assessment procedures.
2. To develop standards and procedures for using external cues and walking aids during assessments.
3. To examine psychometric properties of recommended assessments.

## Methods

### Organisation and participants of the expert panel

The international expert panel was organised by, and held at the Karlsruhe Institute of Technology (KIT) at the Institute of Sports and Sports Science (Karlsruhe, Germany). It was composed according to two main aspects: interdisciplinary variety and practical relevance. Interdisciplinarity and methodological variety was achieved by inviting researches from motor and cognitive sciences as well as the humanities, social, and natural sciences. Practical relevance was obtained by involving researchers with direct experience in the development and evaluation of motor assessments or with experience in dementia research. The initial group invited to this expert panel comprised 27 international researchers, who were chosen based on existing relations with the KIT and a literature screening aimed to identify researchers located within geographical proximity who were interested in motor assessments in IWD.

The expert panel consisted of two interrelated one-day meetings, aiming to achieve an iterative structure. At the first meeting in December 2014, the participants were twelve researchers from five institutions in two countries (Germany and Australia) in the disciplines sports science (especially focusing on biomechanics, human movement science, motor control and learning, sports psychology, sports therapy, and training science), movement and sport gerontology, and psychology. The second meeting in February 2015, comprised a group of five researchers from Germany and the Netherlands covering the disciplines sports science (especially focusing on biomechanics, human movement science, sports therapy, and training science), as well as movement and sport gerontology. With one exception, all researchers of the second meeting also participated in the first. More information on participating researchers is given in the declaration section (see Authors’ information).

### Discussion and guiding questions during the expert panel

Prior to the first meeting, the host institution (KIT) elaborated guiding questions based on challenges in assessing motor performance in IWD identified by literature review. These questions included aspects of appropriateness and standardisation of motor assessments used in previous trials with IWD, use of external cues and walking aids during assessments, and psychometric properties of the considered tools (see Table 1). To enable these guiding questions to be discussed, the KIT research group presented an introductory overview of current research practises and participating researchers introduced their own research experiences with IWD at the first meeting. The discussion was stimulated by the research team of the host institution and one participating researcher supported its guidance.

**Table 1:** Guiding questions for the discussion during the expert panel

	Guiding questions
Appropriateness and standardisation	Are existing motor assessments appropriate for investigating motor performance in IWD? Which motor assessments can be recommended for IWD? How can these motor assessments be standardised?
Use of external cues and walking aids	Should the use of external cues during motor assessments in IWD be allowed or not? If yes, which kind of external cues should be allowed? How can external cues be standardised? Should the use of walking aids be allowed during motor assessments in IWD or not? If no, what if IWD are not able to perform task without? If yes, which kind of walking aids should be allowed?
Psychometric properties	Are existing motor assessments valid and reliable to investigate motor performance in IWD?

*IWD* Individuals with dementia

The first meeting focused on the appropriateness of available motor assessments. In a first step, specific characteristics of IWD and derived demands for motor assessments were considered. Subsequently, these demands were applied to motor assessment tools used in previous trials, which were identified in randomised controlled trials initially analysed for a systematic review assessing effects of physical activity on motor and cognitive performance in IWD [32], supplemented by studies of Bossers et al. [33–35] (see Table 2). Each tool was then rated whether it sufficiently considered specific characteristics of IWD or could be tailored to them. Specific criteria that impacted this evaluation were duration, instructions, and complexity of each assessment tool, as well as physical strains caused in participants. To address standardisation, descriptions and variations of identified motor assessments were considered and possible modifications were discussed. The discussion concerning the use of external cues and motor assessments focused on the two contrary points of view identified in literature: their need to ensure feasibility and safety vs. the influence of external cues and walking aids on the results of motor assessments. Finally, psychometric properties of the identified motor assessments were considered based on available investigations.

**Table 2:** Selection of motor assessments^a^ discussed in the expert panel

Assessments	Outcomes assessed in previous trials	Description
Balance		
*Static balance assessments*		
One-leg balance test [36]	Balance [68]	Standing on one leg while participants’ ability to maintain this stance for 5 s is recorded.
Single leg stance	Lower leg strength and balance [69]	Standing on a single leg alternately for 60 s with both eyes open and closed while time is recorded.
Frailty and Injuries: Cooperative Studies of Intervention Technique - subtest 4 [37]	Balance, static balance [33, 34]	Performing four different stances for 10 s while participants’ ability to maintain these stance is evaluated: (1) feet together, (2) semi-tandem, (3) tandem, (4) single-leg.
Posturography platforms assessing postural sway	Balance [70]	Standing quietly on a posturography platform for 60 s with eyes open while elliptical area covered by moving centre of gravity is recorded.
Functional Reach Test [38]	Balance and stability [71]	Standing next to a wall, holding one arm parallel to a metre stick attached to the wall at shoulder height, and reaching forward as far as possible without losing balance or changing foot position, while distance from starting to end position is recorded.
*Dynamic balance assessments*		
Figure of Eight Test [39]	Balance, dynamic balance [33, 34]	Walking a lap of a standard figure-eight trajectory as quickly and accurately as possible while walking speed and number of oversteps are recorded.
Groningen Meander Walking Test [27]	Balance, dynamic walking ability [34]	Walking over a meandering curved line as quickly and accurately as possible while time and number of oversteps are recorded. Use of a walking aid is allowed.
*Balance scales*		
Berg Balance Scale [40]	Functional balance, balance impairment [69, 72, 73]	14-item functional balance assessment with simple everyday tasks (reaching, bending, transferring, standing and rising) which are graded on a five-point ordinal scale (0 to 4).
Performance Oriented Mobility Assessment [41]	Gait and balance [74, 75]	Scale with two parts, assessing balance (sitting balance, rising from a chair and sitting down, standing balance with eyes open then closed, and turning balance) and gait (gait initiation, step length and height, symmetry, continuity, path direction, and trunk sway).
Mobility and gait		
*Get up & go tests*		
Get-Up and Go Test [44]	Not specified [68]	Standing up from a chair, walking 3 m, turning around, walking back to the chair, and sitting down, while performance is evaluated from 1 to 5 (1 = no instability to 5 = very abnormal). Use of a walking aid is allowed.
Timed Up & Go Test [47]	Mobility, functional mobility, balance, dynamic balance, locomotion, muscle-nerve coordination, agility [33, 34, 70–74]	Standing up from a chair, walking 3 m, turning around, walking back to the chair, and sitting down, while time is measured. Use of a walking aid is allowed.
8-ft up-and-go test [54]	Speed, agility, and balance while moving [75]	Standing up from a chair, walking 8 ft, turning around, walking back to the chair, and sitting down, while time is measured.
Manual Timed Up & Go Test [76, 77]	Mobility [73]	Timed Up & Go Test with additional manual task (carrying a glass of water).
Cognitive Timed Up & Go Test [77]	Mobility [73]	Timed Up & Go Test with additional cognitive task (counting backwards by threes).
*Walk tests / instrumented gait analysis*		
6-m walk test [45]	Mobility, walking speed [33, 34, 68]	Walking 6 m with comfortable pace while time is recorded. Use of walking aid is allowed.
10-m walk test [45]	Walking speed [70]	Walking 10 m with comfortable pace while time is recorded. Use of walking aid is allowed.
Instrumented gait analysis [46, 78]	Walking speed, stride length, double limb support time [79]	Walking at a comfortable pace over an electronic walkway while spatiotemporal gait parameters are recorded.
Strength		
*Sit-to-stand tests*		
Five Times Sit-to-Stand Test [52]	Lower extremity muscle strength and muscle endurance [71, 74]	Performing five repetitions of the sit-to-stand task without upper extremity assistance while time is recorded.
30-s chair-stand test [55]	Muscle dynamic strength endurance of legs [75]	Performing as many repetitions of the sit-to-stand task as possible within 30 s with arms folded across chest.
Modified 30-s chair-stand test, use of upper limbs allowed [20, 55]	Lower body strength, leg strength [33, 34]	Performing as many repetitions of sit-to-stand task as possible within 30 s with upper extremity assistance.
Stair-climbing performance [53]	Functional performance [74]	Climbing a flight with 13 stairs while time is recorded.
*Instrumented assessments*		
Maximal isometric strength assessed with dynamometers [80]	Maximal isometric muscle strength, maximal knee extension strength [33, 34, 74]	Pushing as hard as possible against the dynamometer after adopting a standardised position while maximum strength and integral over time are recorded.
One-repetition maximum in leg press	Maximal dynamic concentric muscle strength in hip and knee extensors [74]	One-repetition maximum as achieved in the leg-press training machine.
*Upper limbs strength*		
Handgrip dynamometer	Handgrip strength [74]	Putting maximum force on a dynamometer while maximal handgrip strength is recorded.
Arm curl test [54]	Muscle dynamic strength endurance of upper body [75]	Performing as many biceps curls as possible within 30 s holding a hand weight of 5 pounds (women) / 8 pounds (men).
Endurance		
*Walk tests*		
2-min walk test [61]	Ambulation [81]	Walking for 2 min while distance is recorded. Use of usual walking aids is allowed.
6-min walk test [62]	Walking performance [82]	Walking for 6 min without use of walking aids while distance is recorded.
Modified 6-min walk test, use of walking aids allowed [23]	Walking endurance, functional mobility [33, 34, 83]	Walking for 6 min while distance is recorded. Use of usual walking aids / physical assistance is allowed.
2-min step test [54])	Aerobic endurance [75]	Performing as many full steps as possible within 2 min, raising knees to a point midway between the patella and iliac crest.
*Flexibility*		
Chair sit-and-reach test [54]	Flexibility, flexibility of lower body [70, 75]	Stretching one leg keeping heel on the floor and trying to touch the toes with the fingers while sitting on a chair while distance between the fingers and toes is recorded.
Back scratch test [54]	Flexibility of upper body [75]	Reaching over the shoulder with one hand and up the middle of the back with the other hand while the distance between extended middle fingers is recorded.
*Functional performance*		
(Modified) Short Physical Performance Battery [63]	Functional performance [74]	Assessment battery with three subtests including standing balance (tandem, semi-tandem, and side-by-side stands), walking speed over an 8-ft walking course, and Five Times Sit-to-Stand Test, which are graded on a five-point ordinal scale (0 to 4). The modified version comprises two subtests including the Five Times Sit-to-Stand Test and gait performance (maximum walking speed, step frequency, cadence).
Senior Fitness Test [54]	Functional capacity [75]	Assessment battery including (1) 30-s chair stand and arm curl test, (2) chair sit-and-reach and back scratch test, (3) 8-ft up-and-go test, and (4) 2-min step test
Physical Performance Test [53]	Performance based motor function activities of daily living [35]	Assessment battery with seven items (writing a sentence, simulated eating, lifting a book onto a shelf, putting on a jacket, picking up a coin from the floor, walking 50 ft, and turning 360°), which are scored on a 4-point Likert scale.
Erlangen Test of Activities of Daily Living [64]	Performance based activities of daily living [35]	Assessment battery with five items (pouring a drink, spreading and cutting a sandwich, opening a small cupboard with a key, washing hands, and tying a bow on a present), which are rated according to correctly performed substeps (0–6 points).

^a^Motor assessments displayed in Table 2 were identified in trials initially analysed for a review assessing effects of physical activity on motor and cognitive performance in IWD [32], supplemented by studies of Bossers et al. [33–35]

The discussion of all guiding questions was gradually specified from open brain storming to personal estimations and final feedback rounds. Thereby, advantages and disadvantages of individual assessment tools and general procedures were gathered and discussed. In doing so, the first meeting elaborated a comprehensive decision basis for the second meeting and developed specific questions for each assessment tool.

Based on the results of the first meeting, the second meeting aimed to derive specific recommendations in a smaller group setting. It started with a summary of the results, which were then examined based on common research practice and own research experiences. In the next step, guiding questions were again critically reflected and specific questions for each assessment tool were discussed in detail. As described above, the discussion again was gradually specified. Finally, the appropriateness of assessment tools and use of external cues and walking aids was established by voting. Consensus was defined as an 80% majority. If consensus was not directly reached, another discussion round was started.

## Results

The discussion on current research practices and experiences resulted in a consensus for applying a sequential approach to assess motor performance in IWD. This means that the level of difficulty is gradually increased, starting with simple and proceeding to more complex tasks, if possible. Such an approach takes specific characteristics and needs of the target population into account, and also considers their heterogeneity and reduced physical capacity. This guarantees appropriate requirements tailored to individual performance and improves the feasibility of assessments and comparability of results. For the further discussions, this sequential approach was supposed to be a basic assumption.

### Recommendations for appropriateness and standardisation of motor assessments

Estimating the appropriateness of motor assessments was performed separately for different physical domains: balance, mobility and gait, strength, endurance, flexibility, and functional performance. To ensure a clear understanding of assessments, short descriptions are given in Table 2.

Based on several underlying considerations extensively described in the following sections, the discussion resulted in recommending the motor assessments summarised in Table 3. As the selection of outcomes and assessments depends on specific objectives and the framework of investigations or aims of clinical examinations, we do not recommend a fixed assessment battery but rather propose a possible selection. Hence, several alternatives are given in Table 3. When composing an assessment battery, it is of great importance to consider the limited capacity of IWD. Thus, we advise restricting the maximum duration of assessments, including rests, to 60 min.

**Table 3:** Recommended motor assessments

Outcome	Assessments
Balance	Frailty and Injuries: Cooperative Studies of Intervention Technique - subtest 4 [37] Groningen Meander Walking Test [27] If the investigation/clinical examination focuses on balance: Berg Balance Scale [40] or Performance Oriented Mobility Assessment [41]
Mobility and gait	Timed Up & Go Test [47] 6-m walk test [45] Instrumented gait analysis (GAITRite®, comfortable pace, single and dual tasks) [46]
Lower limb strength	Modified 30-s chair-stand test [20, 55], including time for five repetitions
Endurance	With constraints, if endurance is an important outcome: 2-min walk test [61] or 6-min walk test [62]
Functional performance	Short Physical Performance Battery [63] Physical Performance Test [53]

#### Balance assessments

To investigate balance, previous trials applied static (one-leg balance test [36], single leg stance, Frailty and Injuries: Cooperative Studies of Intervention Technique - subtest 4 (FICSIT-4) [37], posturography platforms, and Functional Reach Test [38]) and dynamic approaches (Figure of Eight Test [39] and Groningen Meander Walking Test (GMWT) [27]). They also utilised balance scales (Berg Balance Scale [40] and Performance Oriented Mobility Assessment [41]) encompassing both static and dynamic tasks.

To assess static balance, we recommend the FICSIT-4. Compared to one-leg balance tests, this assessment fulfils the requirement of a sequential approach, starting with a less demanding postural position (parallel stance) which is gradually increased (semi-tandem, tandem, and one-leg stance). Assessment tools recording postural sway, like posturography platforms or force plates, can provide more precise information on static balance. However, many of these assessments cannot be recommended for IWD, since they overtax their physical and balance capabilities. In this context, Ruhe et al. [42] performed a systematic review considering various ages and health groups and suggested that three to five repetitions of 90 s each are necessary to reach acceptable reliability values for centre of pressure sway measures. Based on our own experiences, we thus queried feasibility for the majority of IWD. The same applies for the Functional Reach Test. Assuming a non-satisfactory execution, like not leaning forward as far as possible due to fear of falling [43], we concluded that the Functional Reach Test is not a valid assessment for static balance in IWD.

The Figure of Eight Test and the GMWT both assess dynamic balance by determining speed and accuracy while walking a prescribed course – a figure of eight trajectory and a meandering curved line, respectively. Compared to the Figure of Eight Test, the walking course of the GMWT was simplified and thereby tailored to the specific characteristics of IWD [27]. Therefore, we recommend using the GMWT to assess dynamic balance in IWD.

Compared to single balance assessments, balance scales like the Berg Balance Scale or the Performance Oriented Mobility Assessment include various items predominately focusing on important tasks for everyday life. With small restrictions concerning the tasks of leaning forward and one-leg stance, all items seem to be feasible for IWD. Thus, we consider both balance scales appropriate to use with IWD. Even though these scales evaluate balance more comprehensively than single assessments, their longer duration needs to be considered. Therefore, we recommend balance scales for investigations or clinical examinations focusing on balance, but suggest using single assessments in trials and clinical examinations investigating various physical domains.

#### Mobility and gait assessments

Common mobility and gait assessments in IWD include get up and go tests [44], WT [45], and gait analyses [46]. All of these assessments are of short duration, apply simple instructions, and include familiar tasks from everyday life. Based on these estimations, we recommend using all three types of mobility and gait assessments for IWD. However, different variations of get up and go tests and WT exist why standardisation of assessment procedures is very important.

Regarding get up and go tests, different versions vary concerning scoring methods and walking distances. The TUG, a get up and go test version introduced by Podsiadlo and Richardson [47], allows a quantitative evaluation through timing and is also the most frequently used approach. We therefore recommend using this version of get up and go tests. However, the TUG and other available get up and go test versions consist of various short tasks, which need to be remembered during execution. Thus, the appropriateness of the TUG for IWD is somewhat limited and predominantly applies for IWD in mild stages of the disease or tailored approaches allowing the use of external cues (see below).

There are WT for different walking distances and paces. Considering spatial limitations and relevance for short distance walking situations in everyday life, we recommend assessing walking at a comfortable pace over a course of six metres. Instrumented gait analysis systems, such as GAITRite® (CIR Systems Inc., Franklin, NJ), can be valuable in providing further detailed information on different spatiotemporal gait parameters. Additionally, dual task conditions can reveal interactions between cognition and gait more clearly [48, 49]. Besides GAITRite®, which is widely used and has been successfully applied in IWD [50, 51], other instrumented gait analysis systems also might be appropriate, but rarely have been investigated in IWD.

#### Strength assessments

Concerning strength outcomes, available tools can be classified as lower limb (STS tests [52], stair-climbing performance [53], and instrumented strength assessments) or upper limb strength assessments (handgrip dynamometers and arm curl test [54]).

For lower limb strength, we recommend STS tests, in particular a modified 30-s chair-stand test, which allows the use of armrests [20, 55]. Although STS performance only partly depends on lower limb strength [56, 57] and using armrests reduces knee and hip moments [58], it is a functional task, which is of high relevance for everyday life. Moreover, many IWD show reduced physical capacity, and thus may not be able to perform the task without the use of armrests. Compared to the Five Times Sit-to-Stand Test, which records the time required to perform five repetitions, the modified 30-s chair-stand test counts the number of repetitions within 30 s and fulfils the criteria of a sequential approach by allowing each participant to be rated independently of the number of STS repetitions. Additionally, the time required for five repetitions can be simultaneously assessed for all participants reaching this threshold. However, we do not recommend other lower limb strength assessments without constraints. Although stair-climbing performance is a clinically relevant measure of leg power [59], its feasibility may be compromised by practical (availability of standardised flight of stairs) and safety (risk of falling) reasons. With regard to instrumented strength assessments (e.g. dynamometers, isokinetic tools, fitness machines, or other apparatus assessing weights), it has been suggested that such assessments are generally too complex and impractical for assessing large groups [60]. Moreover, their suitability for IWD is questionable, as task-specific strength assessments are partly based on complex motion sequences, which are not related to everyday motor experiences, and therefore conflict with the decreased ability to develop and perform new or complex motor sequences of IWD [11].

We cannot recommend any of the available assessments for upper limb strength without constraints. Dynamometers assessing handgrip strength were scarcely applied in IWD and first need to prove feasibility. The arm curl test was deemed unsuitable for IWD, because it involves a motion sequence unrelated to everyday life (see instrumented strength assessments).

#### Endurance assessments

The 6- or 2-min WT [61, 62], as well as the 2-min step test from the Senior Fitness Test (SFT) [54] are available for endurance assessments. We do not recommend these assessments without constraints. All assessments require participants to stand or walk for two or six minutes, respectively. In contrast, IWD often suffer from multiple motor impairments, frequently affecting the performance of standing or walking. Thus, available endurance assessments seem unsuitable for IWD. Consequently, we suggest limiting the use of such assessments only if specifically indicated by the study design or aim of clinical examination. Nonetheless, developing novel, feasible endurance assessments for frail IWD and examining feasibility of existing assessments well-established in other populations, such as ergometer tests, are indicated.

#### Flexibility assessments

Only few previous investigations have incorporated flexibility assessments in IWD. Accordingly, the discussion did not consider flexibility assessments in detail. Examples are the chair sit-and-reach test, as well as the back scratch test from the SFT [54]. As information on their feasibility in IWD is scarcely available, we suggest not using these flexibility assessments, unless flexibility is a central outcome measure.

#### Functional performance assessments

Looking at functional performance assessments, previous trials applied the Short Physical Performance Battery [63], the Physical Performance Test [53], the Erlangen Test of Activities of Daily Living [64], and the SFT [54]. Among these, Freiberger et al. [65] recommend both the Short Physical Performance Battery and the Physical Performance Test for unimpaired older adults. Both assessments apply tasks relevant to everyday life (e.g., simulated eating, putting on a jacket, standing up from a chair, and walking), and previous trials have demonstrated feasibility in IWD. Thus, our recommendation is to include both assessments to assess functional performance in IWD.

The Erlangen Test of Activities of Daily Living, specifically developed for IWD, is easy and short to administer and includes tasks demonstrating relevance for everyday life (e.g. pouring a drink or washing hands) [64]. This indicates its appropriateness for IWD. However, it seems to be too easy for individuals with mild dementia [66], and therefore we do not recommend it without constraints. Moreover, we do not recommend the SFT for IWD, because it comprises tasks such as the arm curl, chair sit-and-reach, back scratch, and 2-min step tests that were deemed unsuitable for IWD (for details please see above).

### Recommendations for the use of external cues and walking aids during assessments

Previous trials assessing motor performance in IWD frequently allowed the use of external cues. However, their influence on results has not yet been well-established. Nevertheless, external cues seem to be important to ensure the feasibility of motor assessments in IWD, and are especially necessary for assessments consisting of many short tasks, such as the TUG [15]. In this context, we note the heterogeneity in external cues applied across previous trials and emphasise the need for standardisation for comparability reasons. Thus, we suggest allowing the exact repetition of instructions but no other external cues, if not otherwise indicated in the assessment protocol. Moreover, we advise a careful documentation and reporting of used external cues. This recommendation of allowing a restricted use of external cues contributes to tailoring motor assessments to specific characteristics of IWD, and is a first step towards standardisation, which needs to be further substantiated. However, the use of external cues is not appropriate for assessments determining complex motor-cognitive performance, such as activities of daily living.

Walking aids are frequently required by both older adults and IWD, and assessment protocols do often not indicate how to deal with them [18]. Despite their influence on detecting gait changes over time [18], we recommend allowing the use of walking aids applied in everyday life due to safety reasons and to avoid missing data. This may also increase ecological validity, since IWD who use a walking aid in everyday life would be examined in their daily situation. Whenever possible, however, the TUG should be performed without walking aids. Focusing on standardisation, we further suggest restricting the use of walking aids to commonly used aids (e.g. walkers, canes, and crutches), which does not include personal assistance. We also recommend carefully documenting and reporting the use of waking aids. Addressing comparability between baseline and post assessment values, additional qualitative analyses may be indicated when considerable changes in the use of walking aids occurred.

### Psychometric properties

Only few investigations examining psychometric properties of motor assessments in IWD were available at the time of the two meetings. Thus, there was no profound empirical basis for evaluating psychometric properties of motor assessments, which emphasises the urgent need for further investigations.

Considering available investigations, validity has been examined too scarcely and heterogeneously to draw comprehensive conclusions. As indicated in Table 4, intraclass correlation coefficient values for recommended assessment tools ranged between .57 and .99, reflecting sufficient relative reliability, whereas higher intra-individual variability shows lower absolute reliability. Based on these findings, it was concluded that the above-recommended assessments show sufficient reliability to assess inter-individual differences in cross sectional or controlled intervention studies, but are not suitable for determining intra-individual changes [15, 20–25, 27–30].

**Table 4:** Relative (ICC) and absolute (SEM, MDC_95_) reliabilities for recommended assessments

Recommended assessments		Test-retest reliability^a^
Frailty and Injuries: Cooperative Studies of Intervention Technique - subtest 4 [37]		ICC = .79–.82 [20]
		SEM = .55–.60 points [20]
		MDC_95_ = 1.52–1.66 points [20]
Groningen Meander Walking Test [27]	Time	ICC = .93–.96 [27]
		SEM = 1.93 s [27]
		MDC_95_ = 5.35 s [27]
	Oversteps	ICC = .57–.79 [27]
		SEM = 1.58 oversteps [27]
		MDC_95_ = 4.38 oversteps [27]
Berg Balance Scale [40]		N/A
Performance Oriented Mobility Assessment [41]		ICC = .96 [15]
Timed Up & Go Test [47]		ICC = .76–.99 [20–22, 24]^b^
		SEM = 1.43–3.03 s [20–22]^b^
		MDC_95_ = 2.42-8.07 s [20–22]^b^
6-m walk test [45]	Time	ICC = .92 [24]
	Speed	ICC = .83–.89 [20]
		SEM = .09–.11 m/s [20]
		MDC_95_ = .25–.29 m/s [20]
	Steps	ICC = .80 [24]
Instrumented gait analysis (GAITRite®, comfortable pace, single task) [46]	Speed	ICC = .95–.98 [21, 25, 29]
		SEM = 0.06 m/s [21]
		MDC_95_ = .11–.13 m/s [21, 25]
	Step/stride length	ICC = .97–.98 [25, 29]
		MDC_95_ = 4.15–5.27 / 8.12–10.24 cm [25]
	Step width	ICC = .92–.95 [25]
		MDC_95_ = 1.83–2.23 cm [25]
	Stance/swing time	ICC = .87–.96 [25, 29]
		MDC_95_ = .03–.06 s [25]
	Cadence	ICC = .88–.91 [25, 29]
		MDC_95_ = 7.64–8.13 steps/min [25]
Instrumented gait analysis (GAITRite®, comfortable pace, dual task) [46]		N/A
Modified 30-s chair-stand test [20, 55]		ICC = .79–.88 [20]
		SEM = .83–1.52 repetitions [20]
		MDC_95_ = 2.30–4.21 repetitions [20]
Short Physical Performance Battery [63]		ICC = .88 [28]
Physical Performance Test [53]		ICC = .90 [30]
2-min walk test [61]		N/A
6-min walk test [62]		ICC = .75–.99 [21, 23]^b^
		SEM = 19.57–21.86 m [21]^c^
		MDC_95_ = 39.76 m [21]^c^

*ICC* intraclass correlation coefficient, *SEM* standard error of measurement, *MDC*_*95*_ minimal detectable change with 95% confidence interval

^a^Between-day test-retest reliability, if not otherwise indicated

^b^Between-day and within-day test-retest reliability

^c^Within-day test-retest reliability

## Discussion

The present article conveys a consensus of recommendations for assessing motor performance in IWD, which was reached during an expert panel with two interrelated one-day meetings at the KIT in December 2014 and February 2015. These recommendations focus on the appropriateness and standardisation of motor assessments for IWD, deal with the use of external cues or walking aids, and consider psychometric properties of recommended assessments.

To appropriately address IWD, we recommend using a sequential approach and suggest a selection of eight motor assessments to investigate balance (FICSIT-4 and GMWT), mobility and gait (TUG, 6-m WT, and instrumented gait analysis), lower limb strength (modified 30-s chair-stand test), and functional performance (Short Physical Performance Battery and Physical Performance Test). Moreover, we put emphasis on a standardised assessment procedure to ensure comparability between different trials/clinical examinations and to thereby allow conclusions to be drawn based on sound evidence. Considering standardisation in general, we advise allowing a restricted use of external cues and walking aids, and to carefully document and report their use. Psychometric properties could not be considered in-depth, but available investigations determined sufficient relative reliability for the majority of recommended assessments. These recommendations were primarily elaborated for research but equally can be applied in clinical practice. However, lower absolute reliability needs to be considered when assessing intra-individual changes.

To our knowledge, this is the first article giving comprehensive recommendations for assessing motor performance in IWD using a qualitative approach. The few available investigations also focusing on recommendations of motor assessments in IWD analysed assessments used in previous trials from a quantitative perspective, and did not deal with standardisation of assessment procedures or tailoring assessments to specific characteristics of IWD. For the most part, the assessments recommended in this paper coincide with these recommendations (see Bossers et al. [19] and McGough et al. [31]).

A major strength of the expert panel was the comprehensive and thorough analysis of the appropriateness of motor assessments considering specific characteristics of IWD. Following the expert panel, the recommended assessments were applied in a trial of our own with IWD in mild to moderate stages of the disease [67] and demonstrated feasibility. This is in line with previous investigations successfully utilising these assessments or determining their reliability. Nevertheless, information on psychometric properties in many cases is still insufficient and further research is needed [19]. Furthermore, our own experiences showed that it was not possible to identically adopt assessment procedures common for healthy older adults for IWD. For example, it was necessary to allow external cues in form of repeated instructions. This clearly illustrates the need for tailored versions of existing motor assessments, which first need to be standardised and evaluated. Unfortunately, it was not possible to discuss more recent findings within another expert meeting. Potential biases need to be stated concerning the choice of motor assessments. Despite applying a systematic approach, considered assessments were restricted to those utilised in randomised controlled trials with IWD. Thus, other potentially appropriate assessments may be missing. Additionally, the derived recommendations could be biased by the researchers’ experiences and preferences. Moreover, this article only considers existing assessments used in previous trials, whereby the recommendations only include the most suitable of the available possibilities. Besides investigating psychometric properties of existing assessments and developing tailored standardised versions, which consider specific characteristics of IWD, future research should also focus on developing new assessments specifically for IWD. In summary, the recommendations in this article were thoroughly deduced from existing literature and consider the psychometric properties as much as possible. However, they should be used carefully as it is important to first evaluate them in different studies with IWD and address further questions due to limited evidence.

## Conclusions

This article contributes to giving recommendations on performing motor assessments in IWD. However, these recommendations show a preliminary character and are not able to deal with all existing questions. One main problem is that most assessments applied in previous trials were not developed initially for IWD and are not well-investigated within this target population.

Finally, we indicate the need for further studies investigating common motor assessments for administration in IWD. We further encourage tailoring assessment procedures and evaluating existing motor assessments according to the special characteristics of IWD, and then investigating these adapted versions. Nevertheless, it will be important to develop and investigate specific assessments specifically for IWD, such as the GMWT.

In line with Gonçalves et al. [4], we encourage scientists and clinical practitioners to reach a consensus concerning the use of motor assessments, and to apply a standardised assessment procedure aiming to enhance comparability in the research field and clinical practice. With regard to scientific publications, we therefore ask scientists to give a detailed report on how they perform motor assessments in IWD, as different modifications exist and it is often not clear which has been applied.

All efforts undertaken to develop and apply standardised and reliable motor assessments which are appropriate and meaningful for IWD are important steps to enhance evidence concerning efficacy and effectiveness of physical activity on motor performance in IWD.

## Abbreviations

FICSIT-4: Frailty and Injuries: Cooperative Studies of Intervention Technique - subtest 4
GMWT: Groningen Meander Walking Test
IWD: individuals with dementia
KIT: Karlsruhe Institute of Technology
SFT: Senior Fitness Test
STS: sit-to-stand
TUG: Timed Up & Go Test
WT: walk test, walk tests
